# Identifying the Atomic Layer Stacking of Mo_2_C MXene by Probe Molecule Adsorption

**DOI:** 10.1021/acs.jpcc.1c07577

**Published:** 2021-11-23

**Authors:** Anabel Jurado, Ángel Morales-García, Francesc Viñes, Francesc Illas

**Affiliations:** Departament de Ciència de Materials i Química Física & Institut de Química Teòrica i Computacional (IQTCUB), Universitat de Barcelona, c/Martí i Franquès 1-11, 08028 Barcelona, Spain

## Abstract

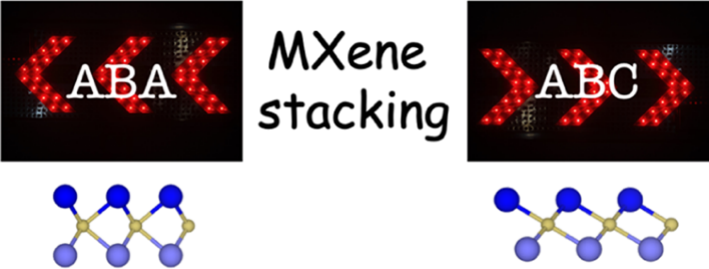

A density functional theory study is presented here aimed at investigating whether the atomic stacking on the new family of two-dimensional MXene materials has an influence on their adsorption properties and whether these properties can provide information about this structural feature. To this end, the Mo_2_C MXene, exhibiting two nearly degenerate crystal structures with either ABC or ABA atomic stacking, is chosen as a case study. The study of the adsorption of CO, CO_2_, and H_2_O on both polymorphs of Mo_2_C reveals substantial differences that could be used in experiments to provide information about the atomic stacking of a given sample. Particularly, we show that the asymmetric and symmetric stretching modes of the adsorbed CO_2_ and the CO stretching mode are clear features that allow one to identify the stacking of atomic layers of the Mo_2_C MXene. The present finding is likely to apply to other MXenes as well.

## 1 Introduction

The discovery of low-dimensional transition-metal carbides and nitrides, known as MXenes,^1,2^ has generated great expectation because of the broad number of applications of these materials emerging from their unique electronic, optical, chemical, mechanical, catalytic, and sensing properties.^3−8^ To some extent, these properties can be modulated by varying the MXene structure and composition. MXenes are specified by the M_*n*+1_X_*n*_T_*x*_ general formula where *n* = 1–3 and M, X, and T_*x*_ denote an early transition metal, carbon or nitrogen, and surface terminations on the surface of the outer transition metal layer, respectively. MXenes are synthesized from MAX precursors, a well-known class of layered materials.^3^ This generally implies a top-down approach where the atomic A-layer—generally a *p*-block element—is selectively etched and MXene flakes of different sizes are thus obtained.^1^ Depending on the synthesis procedure and conditions, the MXene flakes can be covered (*i.e.*, functionalized) by O, H, OH, NH, F, Cl, Br, S, Se, or Te,^6,9,10^ although terminations can be altered or even completely removed by postprocessing.^10−12^ The resulting MXenes may have different stoichiometries depending on the occupation of M sites, which may correspond to one or more transition metal atoms forming solid solutions or ordered structures. In the first case, one has the conventional family of MXenes, whereas the second case leads to newer families that are referred to as *i*-MXenes or *o*-MXenes.^13−15^ Recently, it has been theoretically suggested that the MXene synthesizability is related somehow to the exfoliating energy of the MAX precursor.^16^ As expected, the MXene atomic composition largely defines the underlying chemistry. For instance, the response of M_2_C and M_2_N MXenes to the presence of carbon dioxide (CO_2_) is larger for M = Ti, Zr, and Hf, milder for M = Cr, Mo, and W, and quite reactive for M = V, Nb, and Ta. Apart from the MXene composition and, obviously, from surface functionalization, there are two additional features that, in principle, can influence the reactivity of a given MXene. These are the number of atomic layers and the atomic layer stacking.

In the case of bare MXenes, the effect of the atomic thickness has been recently investigated, analyzing the adsorption of CO_2_ over a broad family of MXene carbides with three, five, or seven (*n* = 1–3) atomic layers.^17^ This computational study confirmed that the thickness of bare MXene has a rather little contribution to the reactivity of MXenes. However, the effect of atomic layer stacking deserves some separate discussion. It is often assumed that because of their good thermal stability,^18^ MXenes feature the ABC stacking inherited from the MAX precursor. In this stacking, each atomic layer is horizontally shifted with respect to the immediate predecessor layer, but a different ABA stacking is also possible. Recently, first-principles based calculations have been used to explore the relative stability of the ABA and ABC stackings for a series of MXenes.^19^ This study predicted that the ABA layer stacking is energetically favorable in Cr-, Mo-, and W-derived MXene carbides and nitrides, and such trends are more pronounced with increasing thickness. Hence, even if the ABC stacking is initially expected to show up, a phase transformation is indeed possible, driven simply by thermodynamics. In addition, the presence of adsorbates could also change the relative stability order of the two phases. This hypothesis has been confirmed in Mo_2_N and W_2_N MXenes, where the activation of the N_2_ molecule promotes somehow the mentioned structural distortion.^20^ Another interesting case is V_2_N MXene, which after etching the MAX precursor initially exhibits the ABC stacking. However, the ABA stacking has also been observed after exposing the former carbides to ammonia.^21^ To study the implication of the stacking on the chemical activity, the adsorption and dissociation of molecular N_2_ were studied, and it confirmed that stacking affects the adsorption strength with changes of up to ∼1 eV.^19^

Previous studies call for further investigations aimed at better understanding the effect that atomic layer stacking in MXenes has on their surface properties and, in particular, on the activation of the stable molecules. Furthermore, there is a need to provide a simple way to assess whether the ABC or ABA stacking is present in a given sample. In the present work, we investigate the adsorption of CO_2_, CO, and H_2_O molecules, taken as probe molecules, on the Mo_2_C MXene which is an appropriate case example. In this MXene, both ABC and ABA stackings are energetically competitive; the ABA being energetically more favorable by ∼0.4 eV per formula unit only. In addition, Mo_2_C is one of the MXenes with moderate adsorption strengths which make it suitable for sensing purposes.^4^ The analysis of the results presented in this work provides compelling evidence that ABC and ABA stackings lead to different chemistries. In addition, we will show that the vibrational frequencies of the adsorbed species provide a simple and efficient way to identify the atomic stacking in the experiments.

## 2 Computational Details and Models

**Figure 1:**
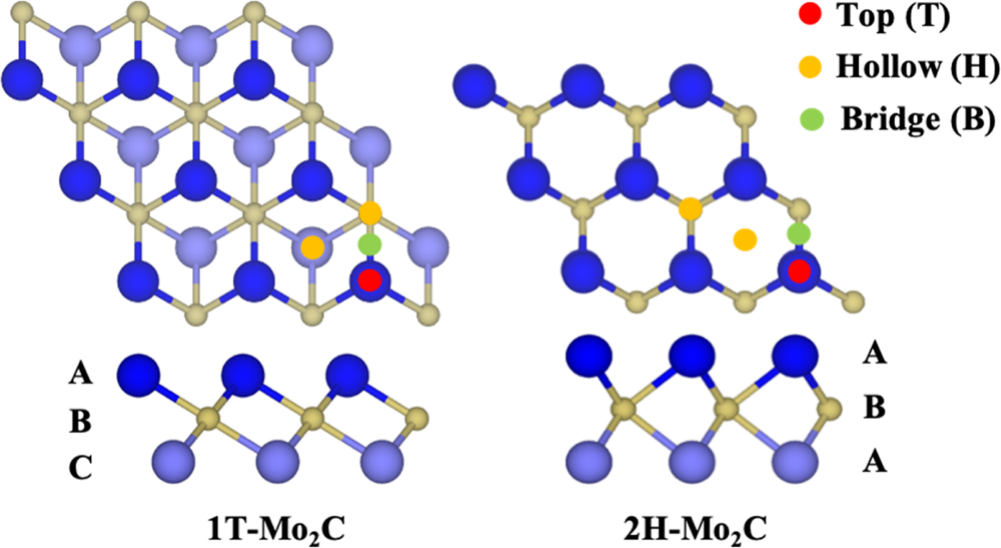
Top (top images) and side (bottom images) views of Mo_2_C(0001) MXene with ABC (1T) and ABA (2H) atomic sequences. Dark and light blue spheres represent the top and bottom Mo layers, while yellow spheres represent the C layer located between them. Red, yellow, and green spheres indicate the adsorption sites where the probed molecules are anchored.

To investigate the influence of the stacking on the surface properties, we have chosen the CO molecule which is a prototypal probe molecule in surface science and included CO_2_ and H_2_O molecules because they exhibit strong interactions with the bare MXene surfaces.^22−24^ The present study relies on periodic density functional theory based calculations for the interaction of CO_2_, CO, and H_2_O on slab models of Mo_2_C MXenes with ABC and ABA stackings. In analogy to single-layer transition metal dichalcogenides featuring similar structures,^25^ these are referred to as 1T and 2H. In the 2H phase, the Mo atomic layers are vertically aligned, whereas in the 1T phase, the two Mo layers are horizontally shifted relative to each other; see Figure 1. A *p*(3×3) supercell is always used to minimize the lateral interaction between adsorbed molecules in the periodically replicated images, and a vacuum width of 10 Å is included to avoid spurious interactions between the periodic replicas in the direction perpendicular to the surface. These settings have proven to be sufficient to obtain numerically converged results (see *e.g.*, the review in ref (26)).

The valence electron density is expanded in a plane wave basis set with a cutoff of 415 eV for the kinetic energy, while the effect of the atomic cores on the valence electronic density is taken into account through the projector augmented wave approach.^27^ A Monkhorst–Pack^28^ grid of 5×5×1 special **k**-points is used to carry out the numerical integrations in the reciprocal space. The total energy is obtained by solving the Kohn–Sham equations with the generalized gradient approximation for the exchange and correlation density functional using the form proposed by Perdew–Burke–Ernzerhof (PBE)^29^ augmented with the Grimme D3 method to account for the contribution of dispersion.^30^ Regarding the choice of the functional used in the present work, it is necessary to point out that none of the existing functionals is free of limitations, so it cannot be claimed that a particular choice will provide near-exact results. Nevertheless, the PBE functional has proven to be among the most robust when describing the properties of bulk and surface transition metals.^31−33^ Consequently, it has been broadly used in the computational heterogeneous catalysis field.^26^ In addition, the adsorption and, more importantly, reactivity of molecules adsorbed at the MXene surfaces is well-described by PBE-D3 as shown in previous works.^17,19^ In any case, we must emphasize that our goal here is to capture trends based on adsorption strengths that allow us to distinguish different stackings of Mo_2_C MXene as discussed later, and the choice of a different functional within the same or higher level of theory is likely to predict essentially the same trends.

The geometry optimization calculations are considered converged when the forces acting over the nuclei are all below 0.01 eV Å^–1^. Overall, this computational setup ensures converged results up to 1 meV in the calculated adsorption energies. For the studied molecules, the adsorption energy, *E*_ads_, on each of the two models of the Mo_2_C(0001) surface, see Figure 1, is computed as
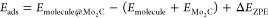
1where *E*_molecule@Mo_2_C_ corresponds to the total energy of the molecule adsorbed on the Mo_2_C surface, while *E*_molecule_ and *E*_Mo_2_C_ stand for the total energy of the isolated molecule in the gas phase and the relaxed pristine Mo_2_C slab model, either with 1T or 2H stacking. Finally, Δ*E*_ZPE_ stands for the difference in the zero-point energy (ZPE) between the gas phase and adsorbed molecules. Note that inclusion of the ZPE term is necessary to compare with the experiments as it accounts for the contribution of the adsorbate normal modes to the total energy. Δ*E*_ZPE_ is here approximated assuming harmonic frequencies for adsorbate vibrations decoupled from surface phonons. The frequencies are obtained by diagonalization of the corresponding block of the Hessian matrix with elements computed as finite difference of analytical gradients with displacements of 0.03 Å. The definition of *E*_ads_ above implies that negative values correspond to exothermic adsorptions. All calculations have been carried out using the Vienna *ab initio* simulation package.^34^

## 3 Results and Discussion

We start this section by analyzing the adsorption strength of CO_2_, CO, and H_2_O species on the two different Mo_2_C(0001) surfaces corresponding to the two possible stackings in this MXene plus a third set corresponding to intermediate structures that are used to extract additional information, as described below. The first surface model, hereafter denoted as 1T-Mo_2_C MXene, corresponds to the ABC stacking expected from the exfoliation of the corresponding MAX phase. The second structural model is obtained by inducing a biaxial in-plane compression on 1T-Mo_2_C; this will be referred to as the strained 1T′-Mo_2_C MXene model. Finally, the third surface is obtained by shifting one of the Mo layers in 1T′-Mo_2_C leading to the 2H-Mo_2_C MXene with ABA stacking. Note also that a biaxial in-plane tensile strain over 2H-Mo_2_C leads to the strained 2H′-Mo_2_C, which by shifting one of the Mo layers closes the cycle as it leads to the original 1T-Mo_2_C MXene. We note that previous theoretical work suggests that the ABA to ABC transition is achievable at a rather low energy cost.^19^ Note that the strain is brought here to easily identify the connectivity among different Mo_2_C structures rather than just relying on raw values for each structure.

From a structural viewpoint, the conventional 1T- and 2H-Mo_2_C MXenes have different lattice parameters, 9.29 and 8.52 Å, respectively. On the other hand, the 1T′-Mo_2_C model has the same stacking of 1T structure but with the 2H-Mo_2_C lattice parameter. Similarly, the 2H′-Mo_2_C features the 2H structure stacking but with the 1T-Mo_2_C lattice parameter. Therefore, the strained 1T′- and 2H′-Mo_2_C MXenes are described as the compressive and tensile structures of 1T- and 2H-Mo_2_C MXenes, respectively. Among them, the 1T-Mo_2_C MXene has been previously investigated by some of us analyzing its adsorption capacity with CO_2_, CO, and H_2_O molecules,^22−24^ and no information is available for the rest of the models. Providing this information is also a goal of the present work.

**Figure 2:**
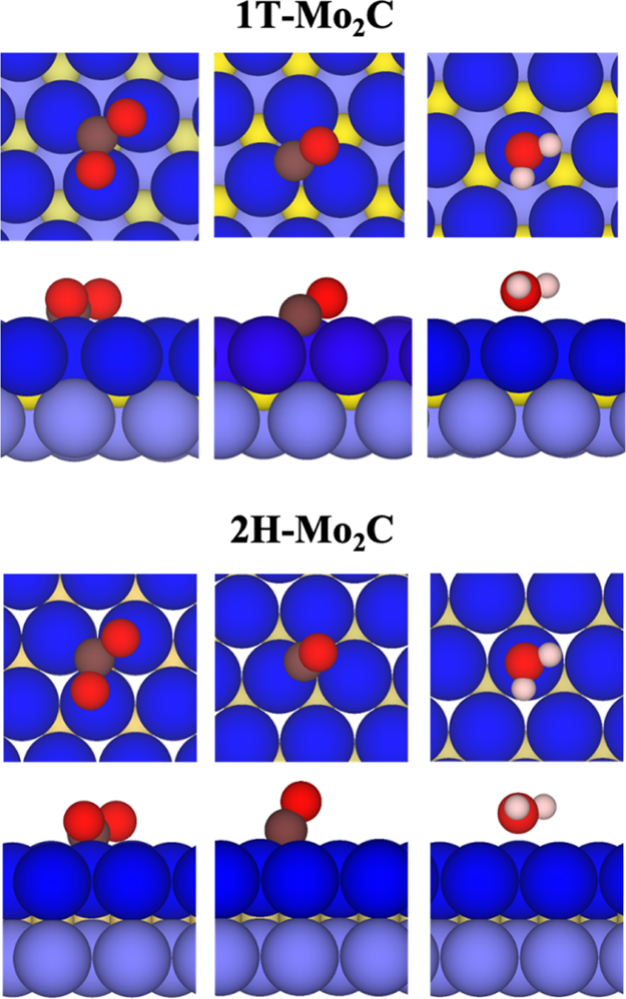
Top (top) and side (bottom) views of the adsorption sites of CO_2_, CO, and H_2_O species on 1T- and 2H-Mo_2_C MXenes. Analogous sites are investigated on 1T′- and 2H′-Mo_2_C MXenes. The sequence color of MXene is described in Figure 1. In addition, the brown, white, and red spheres represent carbon, hydrogen, and oxygen atoms, respectively.

Based on the topology of the MXene surface, we have considered top (T), bridge (B), and hollow (H) sites (see Figure 1), which are systematically investigated for all probed molecules. Furthermore, the probed molecules are anchored over MXene surfaces considering different conformations. Different sites and molecular orientations have been investigated, the most likely sites being those depicted in Figure 2 for the basal (0001) surface of the 1T- and 2H-Mo_2_C MXenes. Here, all these sites are systematically analyzed on the strained 1T′- and 2H′-Mo_2_C MXene surfaces. By this analysis, one the most stable site and molecular conformation are determined.^22−24^ Starting with the CO_2_ molecule, the calculated adsorption energy, structural features, and topological Bader charge are listed in Table 1. The adsorption of CO_2_ is clearly exothermic regardless of the Mo_2_C MXene surface considered. This strong chemisorption promotes the elongation of the C–O distance and the O–C–O angle closure with respect to the gas-phase values. Precisely, the structural deformation of the CO_2_ molecule has a rather large energy cost which is the reason why the adsorption energy is only moderate. Looking at Figure 2, one can observe that the C atom is well-located over a H site, whereas the O atoms are connected to MXenes with Mo atoms locating on top sites. This flat orientation of the CO_2_ molecule promotes the largest adsorption energies in all Mo_2_C MXene substrates investigated here. In addition, there is a considerable net electron transfer from the MXene surface toward the CO_2_ molecule, which thus becomes the activated CO_2_^δ−^ adsorbed species. Following the cycle-like sequence outlined above when describing the surface models, note that the CO_2_ adsorption energy on the 1T-Mo_2_C MXene of −1.80 eV decreases to −1.21 eV on the 1T′-Mo_2_C MXene because of the compression strain. Going to the 2H-Mo_2_C MXene further reduces the adsorption energy to −0.94 eV. Finally, the tensile strain increases, as expected, the activation of the resulting 2H′-Mo_2_C with *E*_ads_ equal to −2.03 eV. In short, the adsorption of CO_2_ depends on the structure of the (0001) Mo_2_C surface, and the elongation of the C–O distances, the O–C–O angle, and the charge of the CO_2_ molecule vary accordingly in a systematic way; see Table 1. The trends for CO_2_ do also hold for the rest of probe molecules, easily interpreted in terms of the relative stability of bare models as explained in detail below.

**Table 1:** Adsorption Energies, *E*_ads_, of CO_2_ on (0001) Mo_2_C MXene Surfaces along with the Most Relevant Structural Features Based on Atomic Distances, *d*, and Angles, ∠^a^

	*E*_ads_/eV	*d*_C–O_/Å	*d*_Mo–O_/Å	*d*_Mo–C_/Å	∠OCO/deg	*Q*/e
1T	–1.80	1.34(×2)	2.08(×2)	1.64	117	–1.27
1T′	–1.21	1.32(×2)	2.11(×2)	1.63	119	–1.24
2H	–0.94	1.31(×2)	2.15(×2)	1.63	121	–1.24
2H′	–2.03	1.32(×2)	2.04(×2)	1.33	115	–1.23
CO_2_ (g)		1.18(×2)			180	

a The topological Bader charge, *Q*, is also displayed. The structural parameters of CO_2_ in the gas phase are included for comparison.

**Figure 3:**
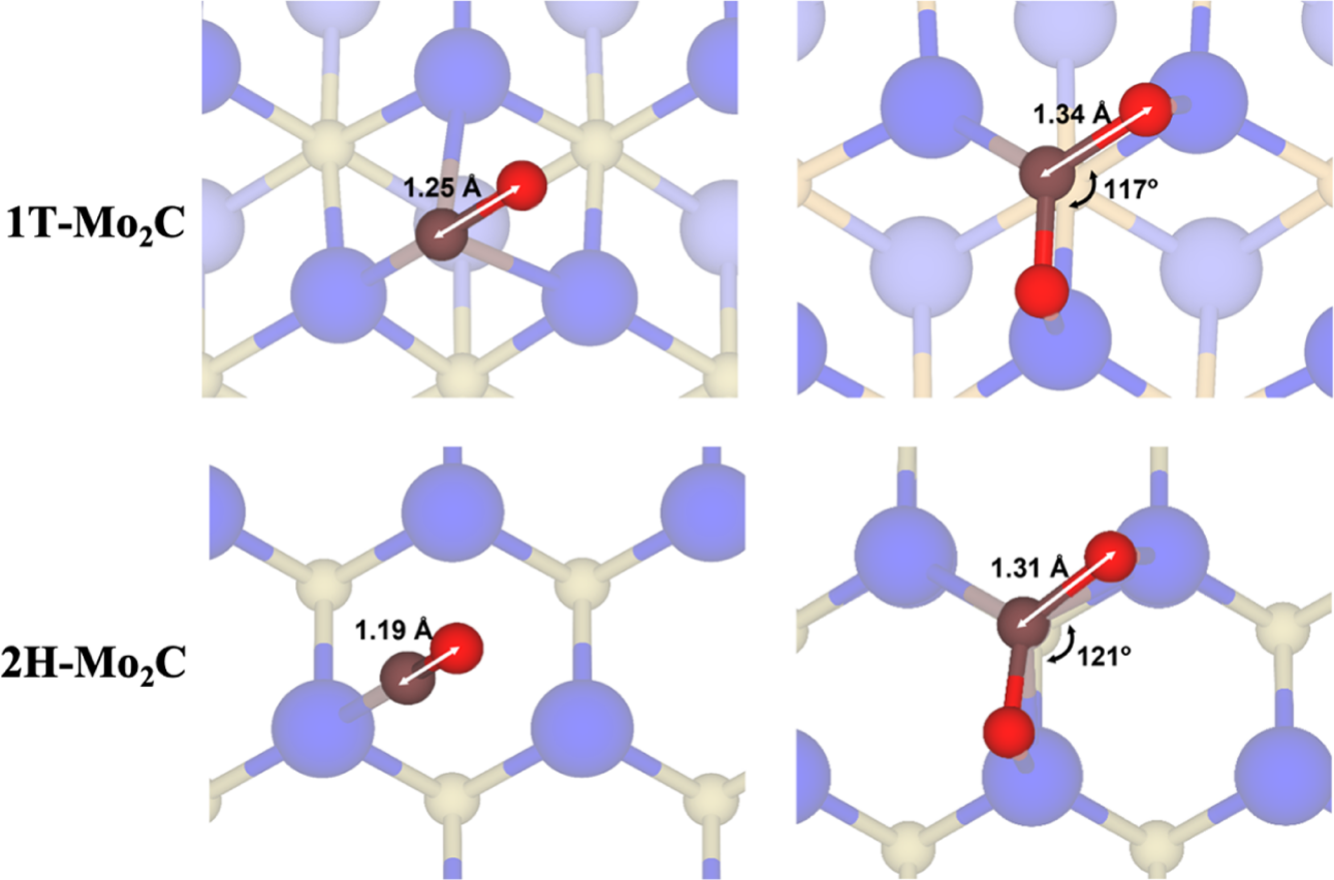
Schematic representation of key structural features of CO and CO_2_ adsorbed on the (0001) 1T- and 2H-Mo_2_C MXene surfaces. MXene atoms are shadowed for better visibility; color code as in Figure 1.

Table 2 compiles the set of results for H_2_O adsorption in the different models. The H_2_O molecule is anchored to the MXene surface on a T site, where the O atom is connected to the Mo atom; see Figure 2. This orientation reports the most favorable adsorption energy. Interestingly, *E*_ads_ decreases along the 1T–1T′–2H structural path and increases along the 2H–2H′–1T path as for CO_2_. However, we note that the adsorption of water could be governed by dispersion because the structure of the H_2_O molecule is almost unaltered showing negligible structural variations with respect to the gas molecule. In addition, the charge transfer toward water is almost zero. Finally, the results for the CO molecule are reported in Table 3. The trends for *E*_ads_ of the CO molecule are once again analogous to those discussed for the CO_2_ molecule; see Table 1. Note, however, that the CO adsorption energy is even larger than that of CO_2_ on the different Mo_2_C MXene surfaces. A plausible reason comes from the largest cost to distort the CO_2_ molecule as depicted clearly in Figure 3. Again, the trend of *E*_ads_ systematically correlates with the C–O bond distance variations with a clear activation of the molecule *via* charge transfer from Mo_2_C MXene surfaces to the CO molecule; see Table 3. Notice that the CO molecule interacts with the Mo_2_C MXene surfaces through the C atom on H sites, adopting a flat-like orientation. Before closing this analysis, an important aspect related to the reactivity of the conventional 1T- and 2H-Mo_2_C MXenes must be pointed out. Clearly, the adsorption strength is larger on the (0001) 1T-than on 2H-Mo_2_C surfaces regardless of the guest molecule. This is directly correlated with the relative stability of the Mo_2_C MXene surfaces; the less stable 1T-Mo_2_C surface gets partially stabilized by adsorption.

**Table 2:** Adsorption Energies, *E*_ads_, of H_2_O on (0001) Mo_2_C MXene Surfaces along with the Most Relevant Structural Features Based on Atomic Distances, *d*, and Angles, ∠^a^

	*E*_ads_/eV	*d*_H–O_/Å	*d*_Mo–O_/Å	∠HOH/deg	*Q*/e
1T	–0.95	0.98(×2)	2.28	106	–0.01
1T′	–0.72	0.98(×2)	2.32	106	–0.03
2H	–0.63	0.98(×2)	2.36	106	–0.03
2H′	–0.68	0.98(×2)	2.35	106	–0.03
H_2_O (g)		0.97(×2)		104	

a The topological Bader charge, *Q*, is also displayed. The structural parameters of H_2_O in the gas phase are included for comparison.

**Table 3:** Adsorption Energies, *E*_ads_, of CO on (0001) Mo_2_C MXene Surfaces along with the Most Relevant Structural Features Based on Atomic Distances, *d*^a^

	*E*_ads_/eV	*d*_C–O_/Å	*d*_Mo–C_/Å	*Q*/e
1T	–2.39	1.26	1.97	–0.97
1T′	–2.08	1.19	2.01	–0.63
2H	–1.83	1.19	2.20	–0.66
2H′	–2.51	1.26	1.99	–1.06
CO (g)		1.14		

a The topological Bader charge, *Q*, is also displayed. The structural parameters of CO in the gas phase are included for comparison.

One of the main conclusions till here is that the stacking of the Mo_2_C MXene influences the adsorption strength and related properties. Thus, one may wonder whether this can be used as an experimental way to identify the stacking of a synthesized Mo_2_C sample. A simple experiment may just involve analyzing the IR or Raman vibrational modes of the selected probe molecules. To this end, Table 4 reports the vibrational analysis of the selected probe molecules including their gas phase and adsorbed configurations on the four (0001) Mo_2_C MXene surfaces. For practical purposes, we focus on the 1T- and 2H-Mo_2_C surfaces, which may be present in experimental Mo_2_C samples based on computational predictions.^19^ The results compiled in Table 4 strongly suggest that CO_2_ and CO are suitable molecules to identify the Mo_2_C stacking based just on the vibrational analysis. Starting with the CO_2_ molecule, its asymmetric stretching emerges as a clear way to identify the Mo_2_C MXene stacking. This vibrational mode of the adsorbed CO_2_ decreases its value by 1000 cm^–1^ with respect to its gas-phase counterpart. More importantly, the asymmetric stretching of the adsorbed CO_2_ molecule on the 1T- and 2H-Mo_2_C MXenes differs by ∼200 cm^–1^. This difference could be in principle sufficient to distinguish ABC and ABA stackings in Mo_2_C MXene samples.

**Table 4:** Vibrational Modes and Frequencies, in cm^–1^, of the CO_2_, H_2_O, and CO Molecules in the Gas Phase and when Adsorbed over (0001) 1T-, 1T′-, 2H-, and 2H′-Mo_2_C MXene Surfaces^a^

	gas phase	1T	1T′	2H	2H′
CO_2_					
ν_as_	2363	1130	1212	1283	1141
Δ		1233	1151	1080	1222
ν_s_	1317	1033	1044	1049	1100
Δ		284	273	268	217
δ	635	662	673	674	710
Δ		–27	–38	–39	–75
H_2_O					
ν_as_	3842	3625	3641	3656	3685
Δ		217	201	186	157
ν_s_	3729	3527	3536	3549	3576
Δ		202	193	180	153
δ	1587	1519	1520	1526	1530
Δ		68	67	61	57
CO					
ν_s_	2131	1465	1795	1773	1439
Δ		666	336	358	692

a The ν_as_, ν_s_, and δ notations correspond to the asymmetric, symmetric stretching, and bending modes. Δ is the difference between gas phase and adsorbed vibrational modes.

Additionally, the CO symmetric stretching can also be used to identify the stacking structure of these MXenes. Upon adsorption on the 1T- and 2H-Mo_2_C surfaces, this mode downshifts by ∼700 and ∼350 cm^–1^ with respect to the gas-phase value, respectively. The difference for the two stacking is ∼300 cm^–1^, sufficient to identify the MXene stacking and, eventually, to see if both are present in freshly synthetized samples. Nevertheless, apart from exhibiting a noticeable shift, a probe molecule for vibrational spectroscopy must be adsorbed in such a way that the corresponding spectroscopic transition is allowed. In the case of adsorbed CO_2_ and CO molecules, the intensity of the corresponding transition fulfils the surface dipole selection rule,^35^ also referred to as the metal surface selection rule,^36^ as there is at least one component of the dipole moment perpendicular to the surface.^37^ This makes these two molecules excellent probe molecules to explore the stacking of MXenes.

## 4 Conclusions

A computational study has been carried out to analyze the effect of the stacking layer of MXenes on the adsorption of molecules, with CO_2_, H_2_O, and CO chosen as examples. Four (0001) Mo_2_C MXene surface models have been considered and labeled as 1T, 2H, 1T′, and 2H′. The first two correspond to ABC and ABA stacking layers, whereas the last two are the compressive (1T′) and tensile (2H′) strains of the former, respectively. We have unequivocally shown that the MXene stacking layer influences significantly the adsorption strength of the CO_2_, H_2_O, and CO molecules which also results in different vibrational shifts with respect to the gas-phase entities. It is suggested that these differences can be used to identify the presence of one, another, or both MXene stackings in the synthesized samples. In particular, the CO_2_ asymmetric and the CO symmetric stretching modes emerge as a rather direct and simple way to identify the stacking of Mo_2_C as both vibrational transitions will carry considerable intensity.

The present results have been obtained for the Mo_2_C MXene, and it is likely that similar conclusions will hold for other MXenes as well. More importantly, this study could be important for experimentalists because spectroscopy measurements would easily identify the MXene stacking layer and observe whether any structural transition takes place when using these materials in practical applications as a sensor or during a given catalytic reaction.
